# Phenolic and Iridoid Glycosides from *Leonurus cardiaca* L. and Their Effects on the α, δ, and γ Subtypes of the PPAR System—Including the Discovery of the Novel Phenylethanoid Cardiaphenyloside A and the Most Active 7-Chloro-6-desoxy-harpagide

**DOI:** 10.3390/molecules30020419

**Published:** 2025-01-20

**Authors:** Kenny Kuchta, Nobuyasu Matsuura, Tung Huu Nguyen, Christian Rusch, Munekazu Iinuma, Yukihiro Shoyama, Hans Wilhelm Rauwald

**Affiliations:** 1 Research Unit for Far Eastern Medicine, Department of Vegetation Analysis and Phytodiversity, Albrecht von Haller Institute of Plant Sciences, Georg August University, u. Karspüle 2, 37073 Göttingen, Germany; 2 Department of Pharmaceutical Biology, Leipzig University, Johannisallee 21, 04103 Leipzig, Germany; 3 Department of Bioscience, Okayama University of Science, Okayama 700-0005, Japan; nobuyasu@ous.ac.jp; 4 Department of Pharmacognosy, Nagasaki International University, Sasebo 859-3243, Japan; tung.nguyenhuu@phenikaa-uni.edu.vn (T.H.N.); oyamash@hotmail.co.jp (Y.S.); 5 Department of Pharmacognosy, Gifu Pharmaceutical University, Gifu 501-1113, Japan; iinumamunekazu@gmail.com

**Keywords:** *Leonurus cardiaca*, *L. japonicus*, phenylethanoid glycosides, iridoid glucosides, PPAR, metabolic syndrome, obesity

## Abstract

*Leonurus cardiaca* L. is known in Europe for its cardioactivity—also in interrelation with known risk factors of the metabolic syndrome—just as *L. japonicus* Houtt. in East Asia; however, up to now, no active constituents could be identified. The three sub-types of PPARs (α, δ, and γ), are involved in controlling the lipid metabolism in the liver and skeletal muscles. Although PPARδ especially is a potential therapeutic target for the metabolic syndrome, insulin resistance, and obesity, no PPARδ agonists with clinical potential have presently been developed. Therefore, nineteen dominant isolated constituents of both species were screened for activity on the metabolic syndrome related PPAR α, δ, and γ in a newly developed luciferase reporter gene assay. Eight phenylethanoid glycosides not previously detected in *L. cardiaca*, including the novel cardiaphenyloside A, as well as the iridoids ajugol and harpagide were found via bioassay-guided isolation and structural elucidation of spectroscopic and chemical evidence. For the PPARδ experiment, all nineteen isolated constituents and GW0742 (positive control) were added to the medium of transfected COS-1 cells and further processed according to a standardized luciferase assay protocol. Only the major iridoid 7-chloro-6-desoxy-harpagide displayed significant activity in the PPARδ assay at 50 μg/mL, while the result for 100 μg/mL was higher than for the GW0742 positive control. Rutin, chicoric acid, and cardiaphenyloside A at 100 μg/mL showed PPARα agonistic activity. For PPARγ, no significant effects were observed. This activity of *Leonurus* extracts and especially of their active constituent 7-chloro-6-desoxy-harpagide on the δ subtype of the PPAR system strongly indicates their potential for anti-obesity therapy.

## 1. Introduction

**Figure 1:**
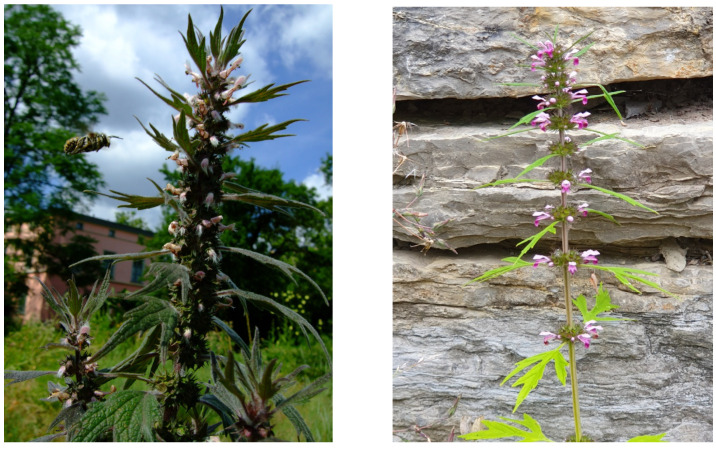
Representative photographs of flowering plants of *Leonurus cardiaca* L. (**left**) and *Leonurus japonicus* Houtt. (**right**); photos: Kenny Kuchta.

European *Leonurus cardiaca* L. (Ph. Eur.) (Figure 1) is traditionally used against tachyarrhythmia, heart failure, and other cardiac disorders for centuries [1,2,3]. Primary and refined extracts of *L. cardiaca* with defined fingerprint HPLC showed cardiac and electrophysiological effects as a mixed I_Ca.L−_, I_Kr_-antagonist, and I_f_ modulator, suggesting its potential as an antianginal and antiarrhythmic medicine [3]. As pointed out by [2], one of the most severe contributors to heart disease is the “metabolic syndrome”. It increases the risks for cardiovascular diseases and type 2 diabetes and may also be included in the cardiac effects of *Leonurus* sp. In the present paper, the fractions of phenylethanoid and iridoid glycosides were preparatively separated and the corresponding structures elucidated. Most interestingly, its Far Eastern pendant *L. japonicus* Houtt. (Figure 1) has been used since earliest times for conditions presently referred to as “metabolic syndrome” [4]. In this context, significant effects of extracts of both *Leonurus* species and the guanidino derivative leonurine, isolated from *L. japonicus*, on the GABA system in a radio-ligand binding assay in vitro have also been reported [5].

Peroxisome proliferator-activated receptors (PPARs) with the sub-types α, δ, and γ bind to the PPAR response element, which is localized in the promoter regions of target genes [6]. PPARα is involved in controlling the lipid metabolism in the liver and skeletal muscles [7]. PPARδ is ubiquitously expressed and is thought to be involved in cell proliferation [8]. Furthermore, it has been shown that PPARδ activation induces the gene expression required for fatty acid oxidation and energy dissipation, thus improving the lipid profile and reducing adiposity [9]. No PPARδ agonists have been developed with clinical potential, although it was recently reported that PPARδ is a potential therapeutic target for metabolic syndrome, insulin resistance, and obesity [10]. PPARγ is strongly expressed in adipocytes and is responsible for adipogenesis. Therefore, nineteen constituents of the two species, including the newly discovered phenylethanoid cardiaphenyloside A, were screened for activity on metabolic syndrome-related PPARs α, δ, and γ in a newly developed luciferase reporter gene assay.

**Figure 2:**
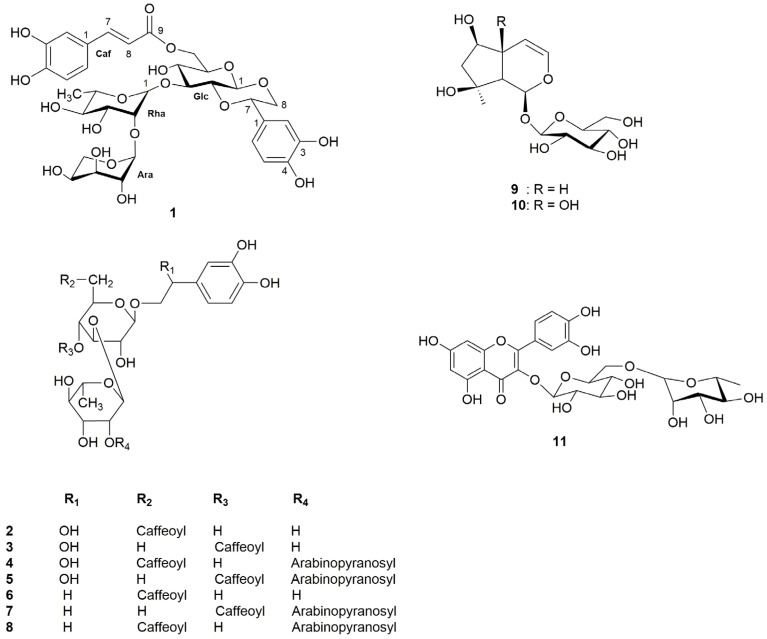
Structures of isolated and elucidated phenylethanoid and iridoid glycosides as well as rutin, which also were examined in PPAR system (For explanation of numbers, see Introduction.).

**Figure 3:**
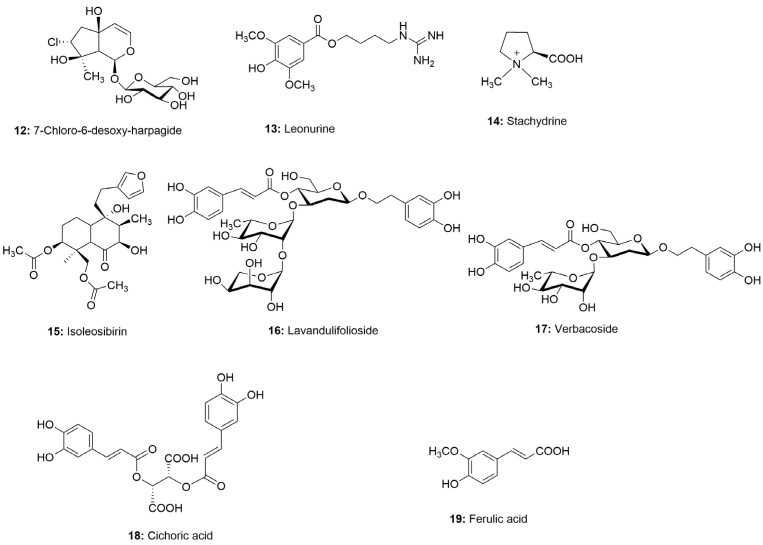
Structures of further phenolic, iridoid and alkaloid constituents from our previous works on *Leonurus spec.* [3,11,14] which were also examined in PPAR system.

Based on HPLC analysis [11], several phenolic components, including phenylethanoid constituents of *L. cardiaca*, namely chlorogenic acid, caffeic acid, ferulic acid, cichoric acid, lavandulifolioside, verbascoside, isoquercitrin, and rutoside, could be detected, isolated, and reliably quantified [3,11] (for structures see Figure 2 and Figure 3).

Our ongoing study on constituents of the aerial parts of *L. cardiaca*, which is generally considered to urgently require a phytochemical re-evaluation [12,13], has resulted in the isolation of eleven glycoside compounds (**1**–**11**), including eight phenylethanoid glycosides not previously found in *L. cardiaca*. In a related study, we reported the isolation of 7-chloro-6-desoxy-harpagide (**12**) from the same plant [14] (Figure 2 and Figure 3). In a preliminary study inspired by its traditional use in East Asia [4], we demonstrated the activity of *L. japonici* herba on the PPAR receptors in the same experimental model. However, no active constituents could be identified among the co-investigated N-containing constituents leonurine (**13**) and stachydrine (**14**) (Figure 2 and Figure 3).

Here, we aim to develop PPAR agonists from potentially active, phenolic, iridoid, and alkaloid constituents of *L. cardiaca* using a newly developed luciferase reporter gene assay [15].

## 2. Results and Discussion

### 2.1. Isolation and Structural Elucidation of Phenylethanoids and Iridoids

Solvent extraction and partition followed by various column chromatography protocols resulted in the isolation of eleven compounds (**1**–**11**) from the aerial parts of *L. cardiaca*. Among the isolated compounds, the known compounds (**2**–**11**) were identified as isocampneoside II (**2**) [16], campneoside II (**3**) [17], 3,4,7-trihydroxy- 8-phenylethoxy-*O*-{[*α*-L-arabinopyranosyl-(1→2)-*α*-L-rhamno-pyranosyl-(1→3)]-6-*O*-caffeoyl-*β*-D-glucopyranoside} (**4**) [18], 3,4,7-trihydroxy-8-phenylethoxy-*O*- {[*α*-L-arabinopyranosyl-(1→2)-*α*-L-rhamnopyranosyl-(1→3)]-4-*O*-caffeoyl-*β*-D-glucopyranoside} (**5**) [18], isoacteoside (**6**) [19], leonoside A (**7**) [19], 3,4-dihydroxy- 8-phenylethoxy-*O*-{[*α*-L-arabinopyranosyl-(1→2)-*α*-L-rhamnopyranosyl-(1→3)]-6-*O*-caffeoyl-*β*-D-glucopyranoside} (**8**) [18], ajugol (**9**) [20], harpagide (**10**) [14,20], and rutin (**11**) [21] on the basis of spectroscopic and physicochemical comparison with those reported in the literature.

**Figure 4:**
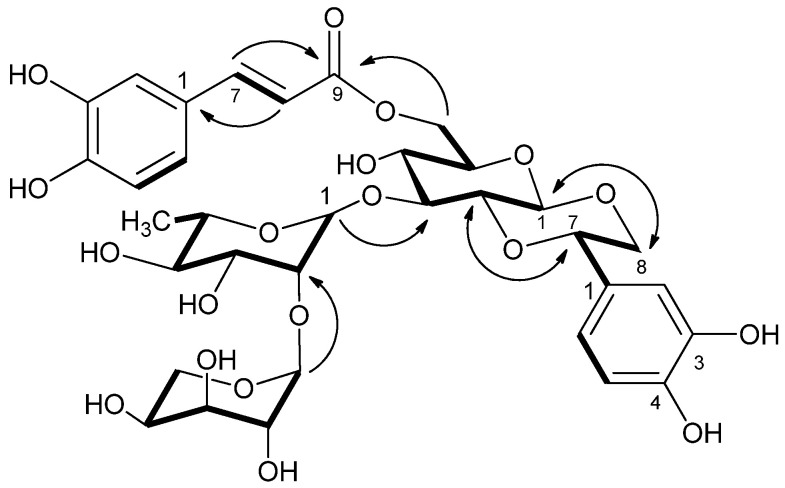
The new compound cardiaphenyloside A (**1**) with key HMBC (arrows) and COSY correlations (bold lines).

Compound **1** was obtained as a white amorphous powder with ${\left[\alpha \right]}_{D}^{20}$ + 26° (*c* 0.15, MeOH). Its molecular formula was determined to be C_34_H_42_O_19_ based on the observed molecular peak at *m/z* 755.2413 [M + H]^+^ (calcd. for C_34_H_43_O_19_, 755.2399) in the HR-ESI-TOF-MS spectrum indicating 14 degrees of unsaturation, as calculated by double bond equivalents. The IR spectrum showed typical absorption bands of OH group (3426 cm^−1^), *α*,*β*-unsaturated ester (1694, 1260 cm^−1^), and aromatic rings (1608, 1516 cm^−1^). The ^1^H-NMR spectrum of **1** showed typical signals of a caffeoyl phenylethanoid glycoside, including two ABX aromatic proton systems [*δ* 6.80 (1H, d, *J* = 1.6 Hz), 6.71 (1H, d, *J* = 8.0 Hz), and 6.69 (1H, dd, *J* = 8.0, 1.6 Hz) and 7.01 (1H, d, *J* = 1.6 Hz), 6.74 (1H, d, *J* = 8.0 Hz), and 6.91 (1H, dd, *J* = 8.0, 1.6 Hz)] and a pair of *trans*-olefinic protons [7,54 (1H, d, *J* = 16.0 Hz) and 6.26 (1H, d, *J* = 16.0 Hz)] [17,18]. (Figure 4) shows the new cardiaphenyloside A (**1**) with HMBC and COSY correlations. Additionally, three anomeric protons at *δ* 5.33 (1H, br s), 4.44 (1H, d, *J* = 8.0 Hz), and 3.90 (1H, d, *J* = 7.6 Hz), which gave correlations in the HMQC spectrum with three anomeric carbon signals at δ 100.6, 99.0, and 107.4, respectively, suggested a triglycoside structure for **1**. The D-glucose, L-arabinose, and L-rhamnose sugar units in the molecule were further determined by HPLC analysis using an amino column after acid hydrolysis of **1**. The configuration of the anomeric protons of the glucose, arabinose, and rhamnose units were assigned as *β*, *α*, and *α* on the basis of their coupling constants [22].

The ^13^C NMR and HMQC spectra of **1** disclosed 34 carbon resonances consisting of 1 methyl, 3 methylenes, 23 methines, and 7 quaternary carbons; of which, the set of nine signals [*δ* 127.7, 115.1, 146.8, 149.7, 116.5, 123.1, 147.3, 114.7, and 169.0] were attributed to the caffeoyl moiety [18]. Further analyses of ^13^C NMR data together with H-H COSY led to discrimination of the signals of each sugar unit (Table 1). The linkage of the sugar chain and partial structures of **1** were confirmed by the HMBC spectrum. The caffeoyl unit was linked to C-6 of the glucose moiety due to a long-range HMBC correlation of H-6_(glu)_/C-9_(caf)_. On the other hand, the arabinose moiety was attached to C-2 of the rhamnose unit, which was found to be fused to the glucose part at C-3 as evidenced by the HMBC cross-peaks of H-1_(Ara)_/C-2_(Rha)_ and H-1_(Rha)_/C-3_(Glc)_, respectively. Taking into account the NMR spectroscopic data and the 14 degrees of unsaturation (see above) calculated from the empirical formula of **1**, it was suggested that **1** had another alicyclic ring except for an α,β-unsaturated ester group, two aromatic rings, one glucose, one rhamnose, and one arabinose. The linkage site of the additional ring was determined according to the obvious HMBC correlations between H-1_(Glc)_/C-8, H-8/C-1_(Glc)_, H-2_(Glc)_/C-7, and H-7/C-2_(Glc)_. The relative stereochemistry of **1** was established by the coupling constants and NOESY correlations. The correlations of H-1_(Glc)_/H-8a, H-1_(Glc)_/H-3_(Glc)_, and H-2_(Glc)_/H-7 suggested that both the glucopyranosyl and alicyclic rings had a chair conformation with trans-fused ring junctions. In the ^1^H-NMR spectrum, the doublet at δ 4.44 (1H, d, *J* = 8.0 Hz) supported that the glucose was of β-conformation, and the large coupling constant (*J* = 10.4 Hz) also indicated that H-7 and H-8a were similar to H-1_(Glc)_ and H-2_(Glc)_ in *trans*-axial orientation [22,23]. All the carbon NMR data and corresponding protons of **1** together with the partial structures were assigned by COSY, HMQC, and HMBC spectra, respectively, and comparison of spectroscopic data with similar phenylethanoid glycosides in the literature [18,23]. Consequently, the structure of **1** was confirmed as shown in (Figure 4) and was named cardiaphenyloside A.

### 2.2. Results and Effects of the 19 Leonurus Constituents on PPAR

**Figure 5:**
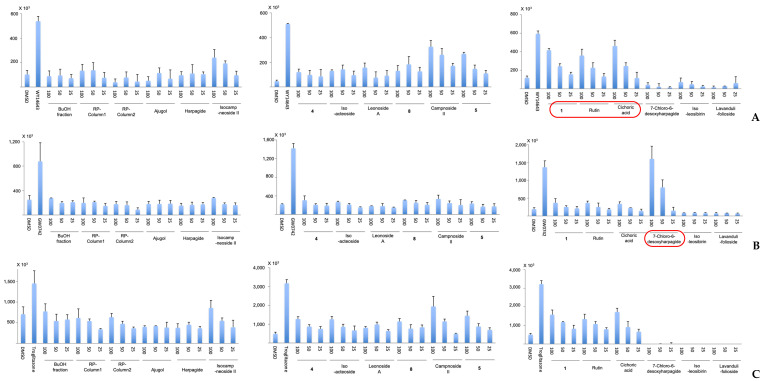
Activation of the PPARα (**A**), PPARδ (**B**), and PPARγ (**C**) systems by isolated constituents of *L. cardiaca*. All experiments were performed in triplicates (*n* = 3) and results given in relative luciferase expression levels (Luciferase/SEAP activity). Statistical analysis was performed with chi-square test, Dennett’s test, and Mann–Whitney’s U-test. Cardiaphenyloside A (**1**) and the compounds (**4**), (**5**), and (**8**) are indicated by their respective numbers.

All 19 described isolated *L. cardiaca* constituents were tested for their activity on all three subtypes of the metabolic syndrome-related PPARs α, δ, and γ in a luciferase reporter protocol [15] (Figure 5). However, the majority of the isolated compounds did not display a significant effect on this target. Only 7-chloro-6-desoxy-harpagide (**12**) was significantly active in the PPARδ assay at 50 μg/mL, while the result for 100 μg/mL was higher than for the GW0742 positive control. Rutin, chicoric acid, and—most interestingly—**1** at 100 μg/mL showed PPARα agonistic activity. For PPARγ, no significant effects were observed for any of the examined compounds. This activity of extracts of medicinal plants of the genus *Leonurus* and especially of their active constituent **12** on the PPARδ subtype of the PPAR system strongly indicates their potential for anti-obesity therapy. However, although the synthetic PPARδ agonist GW501516 attenuates the metabolic syndrome according to two phase IIa clinical trials [24,25], this compound was later found to cause cancer at dosages of 3 mg/kg/day in both mice and rats [26]. Instead of the known risks, a new placebo-controlled human trial on the synthetic GW501516 was published recently [27], in which the authors reasoned that “further research on GW501516 and similar PPARδ agonists are required as some benefits may outweigh impeding risks, especially in the context of metabolic syndrome-like conditions”, thus judging the risk–benefit ratio as positive due to the highly debilitating effects of metabolic syndrome. Nevertheless, no PPARδ agonists have ever been developed with clinical potential until now.

Here, we report that preparations of *L. cardiaca*, a traditional herbal drug, long established as safe and generally available in German pharmacies for single prescription, as well as its active constituent **12** represent an available PPARδ agonist that may be used in the therapy of the metabolic syndrome in accordance with their traditional indication. Furthermore, diabetes has been identified as closely associated with the malignancy of cancers [28], further emphasizing the importance of the presented discovery. *L. cardiaca* and *L. japonicus* extracts may therefore not only be highly useful in the therapy of the metabolic syndrome and associated ailments, but they may also contribute to prevention of much more severe oncological diseases like, e.g., bile duct carcinoma [28]. Interestingly, compound **12**, isolated by a special method according to [14], has till now only been found once in the plant kingdom, namely also in a Lamiaceae, *Physostegia virginiana*, only in the subspecies *virginiana var. speciosa* [29]. In contrast, in our result, it is a major iridoid from *L. cardiaca*. This unusual chlorinated harpagide derivative also represents an interesting new lead in pharmacological research. 

## 3. Materials and Methods

### 3.1. General Procedures

Optical rotations: JASCO-DIP-360 digital polarimeter (JASCO, Easton, MD, USA). NMR: JEOL ECX 400 (JEOL Ltd., Tokyo, Japan). HR-ESI-TOFMS: JEOLAccuTOF^TM^LC 1100 (JEOL Ltd., Tokyo, Japan). HPLC analysis of sugars: Agilent 1100 Series HPLC system (Agilent Technologies, Santa Clara, CA, USA) with a YMC-Pack NH_2_ column (250 × 4.6 mm i.d., NH12S05-2546WT, YMC Co. Ltd., Kyoto, Japan) and an optical rotation detector JASCO OR-2090 (JASCO, Easton, MD, USA). CC: Silica gel 60 (230–400 mesh, Nacalai Tesque Inc., Kyoto, Japan)/YMC ODS-A gel (50 µm, YMC Co., Ltd., Kyoto, Japan). TLC: Kieselgel 60 F_254_ and Silica gel 60 RP-18 F_254S_ (Merck, Damstadt, Germany) plates, spraying with 10% aqueous H_2_SO_4_ solution, followed by heating.

### 3.2. Chemicals

For the isolation and purification of cardiaphenyloside A (**1**), isocampneoside II (**2**), campneoside II (**3**), 3,4,7-trihydroxy-8-phenylethoxy-O-{[α-L-arabinopyranosyl-(1→2)- α-L-rhamnopyranosyl-(1→3)]-6-O-caffeoyl-β-D-glucopyranoside} (**4**), 3,4,7-trihydroxy-8- phenylethoxy-O-{[α-L-arabinopyranosyl-(1→2)-α-L-rhamnopyranosyl-(1→3)]-4-O-caffeoyl-β-D-glucopyranoside} (**5**), isoacteoside (**6**), leonoside A (**7**), 3,4-dihydroxy- 8-phenylethoxy-O-{[α-L-arabinopyranosyl-(1→2)-α-L-rhamnopyranosyl-(1→3)]-6-O-caffeoyl-β-D-glucopyranoside} (**8**), and ajugol (**9**), see the Results Section. Although harpagide (**10**) and rutin (**11**) were isolated during the same procedure, additional commercial samples were purchased for both harpagide (**10**) (>99%/HPLC; Phytolab, Vestenbergsgreuth, purified) and rutin (**11**) (>98%/HPLC; Merck, Darmstadt, purified).

7-chloro-6-desoxy-harpagide (**12**) (at least 97% purity) was isolated from *L. cardiaca* by a special method; the structural elucidation was performed via NMR and MS data [14]. Leonurine (**13**), stachydrine (**14**), and isoleosibirin (**15**) are described in [5]. Pure phenolic standards, lavandulifolioside (**16**), verbascoside (**17**) (>99%/HPLC), cichoric acid (**18**) (>98%/HPLC), and ferulic acid (**19**) (99%/HPLC) were isolated in our lab (see above).

### 3.3. Plant Material

*L. cardiaca* herba: Caelo (Batch No 32054354). Authentic voucher specimens, deposited in the herbarium of the Institute of Special Botany, Leipzig University (EDV registration number 167244).

### 3.4. Extraction and Isolation

*L. cardiaca* herba was extracted according to [3], yielding 55.0 g of refined extract, which was suspended in H_2_O (150 mL) and successively partitioned with CH_2_Cl_2_, EtOAc, and n-BuOH (each 150 mL × 3). The obtained BuOH portion (4.8 g) was subjected to a reversed-phase (RP) column eluting with a mixture of MeOH-H_2_O (1:3, *v*/*v*) to give 12 fractions (fr.1 ~ fr.12). Fr.2 (170 mg) was then rechromatographed on a silica gel column eluting with CHCl_3_-MeOH-H_2_O (5:1:0.1, *v*/*v*/*v*) to afford compounds **9** (15 mg) and **10** (10 mg). Next, fr.8 (205 mg) was further purified by a silica gel column eluting with CHCl_3_-MeOH-H_2_O (7:3:0.4, *v*/*v*/*v*) to yield **2** (40 mg) and **4** (30 mg). Similarly, fr.9 (235 mg) was loaded onto a silica gel column with CHCl_3_-MeOH-H_2_O (7:3:0.4, *v*/*v*/*v*) as the eluent to furnish **6** (13 mg), **7** (14 mg), and **8** (25 mg), respectively. Fr.10 (125 mg) was subjected to a silica gel column with CHCl_3_-MeOH-H_2_O (7:3:0.4, *v*/*v*/*v*), followed by an RP column with MeOH-H_2_O (1:1, *v*/*v*) to yield **1** (21 mg) and **11** (12 mg). Finally, fr.7 (101 mg) was chromatographed on a silica gel column with CHCl_3_-MeOH-H_2_O (6:3:0.5, *v*/*v*/*v*) to afford **3** (13 mg) and **5** (12 mg). Cardiaphenyloside A (**1**) (Table 1): white amorphous powder; ${\left[\alpha \right]}_{D}^{20}$ +26° (*c* 0.15, MeOH); IR (KBr) *ν*_max_ 3426, 1694, 1608, 1516, and 1260 cm^−1^; ^1^H (400 MHz, CD_3_OD) and ^13^C NMR (100 MHz, CD_3_OD) (for data, see Table 1); HR-ESI-TOF-MS (positive ion mode) *m/z m/z* 755.2413 [M + H]^+^ (calcd. for C_34_H_43_O_19_, 755.2399). A solution of **1** (3.0 mg) in 1.0 M HCl (5.0 mL) was heated under reflux for 4 h. After cooling, the reaction mixture was poured into ice water and neutralized with Amberlite IRA-400 (hydroxyl form), and the resin was removed by filtration. Then, the filtrate was concentrated in vacuo to dryness, followed by partition between EtOAc and H_2_O. The aqueous layers after filtration were subjected to HPLC analysis with the mobile phase of CH_3_CN–H_2_O (80:20, *v*/*v*) at flow rate 0.40 mL/min. Identification of sugar components in the aqueous layers was carried out by comparison of their retention time and optical rotation with those of authentic samples. *t*_R_: 7.2 min (L-rhamnose, negative optical rotation), 8.3 min (L-arabinose, positive optical rotation), and 9.6 min (D-glucose, positive optical rotation), respectively. D-glucose, L-rhamnose, and L-arabinose were found from **1**.

### 3.5. Luciferase Reporter Assay for the 19 Leonurus Constituents

For the PPARδ experiment, all 19 isolated constituents (at 25, 50, and 100 μg/mL each) and GW0742 (positive control, 0.1 nM) were dissolved in DMSO and added to the medium of the transfected COS-1 cells (reporter plasmid p17m2G) and processed as described in detail by [15]. An analogous approach was used for PPARα and PPARγ, in which case the COS-1 cells were transfected with pPPARα or γ-GAL4 and p17m2G or positive controls 50 μM WY14643 or 10 μM troglitazone, respectively. For all data, each value represents the mean ± standard error of three experiments. Statistical analysis was performed with chi-square test, Dennett’s test, and Mann–Whitney’s U-test.

### 3.6. HPLC

All extracts of *L. cardiaca* and *L. japonicus* aerial parts were analyzed using an HPLC protocol recently developed by our group and published in [11], where all experimental details are described.

## Figures and Tables

**Table 1:** ^1^H− and ^13^C− NMR data for **1** in CD_3_OD.

Position	*δ*_C_	*δ*_H_
**Aglycone moiety**		
1	130.0	
2	115.4	6.80 (d, *J* = 1.6 Hz)
3	146.4	
4	146.8	
5	116.4	6.71 (d, *J* = 8.0 Hz)
6	119.8	6.69 (dd, *J* = 8.0, 1.6 Hz)
7	78.7	4.50 (dd, *J* = 10.4, 2.0 Hz)
8	72.5	3.93 ^#^; 3.66 (br d, *J* = 12.0 Hz)
**Caffeoyl moiety**		
1	127.7	
2	115.1	7.01 (d, *J* = 1.6 Hz)
3	146.8	
4	149.7	
5	116.5	6.74 (d, *J* = 8.0 Hz)
6	123.1	6.91 (dd, *J* = 8.0, 2.0 Hz)
7	147.3	7.54 (d, *J* = 16.0 Hz)
8	114.7	6.26 (d, *J* = 16.0 Hz)
9	169.0	
**Sugar chain**		
Glucose (Glc)		
1	99.0	4.44 (d, *J* = 8.0 Hz)
2	82.0	3.35 ^#^
3	79.6	3.72 ^#^
4	70.2	3.48 ^#^
5	77.2	3.70 ^#^
6	64.5	4.50 (dd, *J* = 11.6, 2.0 Hz); 4.32 (dd, *J* = 12.0, 5.2 Hz)
Rhamnose (Rha)		
1	100.6	5.33 (br s)
2	83.2	3.74 ^#^
3	71.8	3.66 ^#^
4	74.1	3.28 ^#^
5	69.7	3.92 ^#^
6	17.9	1.17 (d, *J* = 6.4 Hz)
Arabinopyranose (Ara)		
1	107.4	3.90 (d, *J* = 7.6 Hz)
2	72.8	3.43 ^#^
3	74.3	3.30 ^#^
4	69.8	3.48 ^#^
5	66.9	3.25 ^#^; 2.69 (br d, *J* = 12.4 Hz)

Assignments were confirmed by COSY, HMQC, HMBC, and NOESY spectra. ^#^ Overlapped with other signals.

## Data Availability

The original contributions presented in this study are included in the article. Further inquiries can be directed to the corresponding authors.
